# Low-Energy Amorphization of Ti_1_Sb_2_Te_5_ Phase Change Alloy Induced by TiTe_2_ Nano-Lamellae

**DOI:** 10.1038/srep30645

**Published:** 2016-07-29

**Authors:** Keyuan Ding, Feng Rao, Shilong Lv, Yan Cheng, Liangcai Wu, Zhitang Song

**Affiliations:** 1 State Key Laboratory of Functional Materials for Informatics, Shanghai Institute of Micro-system and Information Technology, Chinese Academy of Sciences, Shanghai 200050, China; 2 University of the Chinese Academy of Sciences, Beijing 100080, China

## Abstract

Increasing SET operation speed and reducing RESET operation energy have always been the innovation direction of phase change memory (PCM) technology. Here, we demonstrate that ∼87% and ∼42% reductions of RESET operation energy can be achieved on PCM cell based on stoichiometric Ti_1_Sb_2_Te_5_ alloy, compared with Ge_2_Sb_2_Te_5_ and non-stoichiometric Ti_0.4_Sb_2_Te_3_ based PCM cells at the same size, respectively. The Ti_1_Sb_2_Te_5_ based PCM cell also shows one order of magnitude faster SET operation speed compared to that of the Ge_2_Sb_2_Te_5_ based one. The enhancements may be caused by substantially increased concentration of TiTe_2_ nano-lamellae in crystalline Ti_1_Sb_2_Te_5_ phase. The highly electrical conduction and lowly thermal dissipation of the TiTe_2_ nano-lamellae play a major role in enhancing the thermal efficiency of the amorphization, prompting the low-energy RESET operation. Our work may inspire the interests to more thorough understanding and tailoring of the nature of the (TiTe_2_)_n_(Sb_2_Te_3_)_m_ pseudobinary system which will be advantageous to realize high-speed and low-energy PCM applications.

Non-volatile phase change memory (PCM), as one of the promising candidates, has great potential to serve as a storage class memory (SCM) to mitigate the widened performance mismatch between dynamic random access memory (DRAM) and non-volatile NAND Flash memory^1^,^2^. In the PCM cell, a chalcogenide material, for example, Ge_2_Sb_2_Te_5_, can be switched between the crystalline (c-) and amorphous (a-) phases, corresponding to the SET and RESET states^3^,^4^, respectively. The big resistance contrast of such two states is utilized for storing “0” and “1” data states. The RESET operation refers to an amorphization procedure which melts the c-phase and subsequently quenches it into a-phase by applying a short intense electrical pulse on the PCM cell. Conversely, a longer pulse of lower intensity for SET operation can heat the a-phase to a temperature between crystallization temperature (*T*_c_) and melting point (*T*_m_) to obtain the c-phase. To achieve high density SCM application, scaling capability of the PCM cell is largely limited by its high RESET current or energy^5^ for which the premature degradation of the switching material or the thermal disturbance among nearest cells would happen and cause reliability and endurance issues^6^.

Since the slow SET speed and high RESET power remain to be important limitations for developing DRAM-like PCM, our previous work demonstrated at least one order of magnitude faster SET speed and as low as one-fifth of the RESET current and energy on a Ti_0.4_Sb_2_Te_3_ based PCM cell compared to those of the Ge_2_Sb_2_Te_5_ based cell with the same size^7^. The stable c-Ti_0.4_Sb_2_Te_3_ alloy has the nano-scale phase separated morphology that hexagonal (HEX) Sb_2_Te_3_ and HEX-TiTe_2_ crystals coexist^8^. The triple-layered TiTe_2_ crystal lamellae locate adjacent to the quintuple-layered Sb_2_Te_3_ grains, while there are also some Ti atoms penetrate into Sb_2_Te_3_ lattice by occupying Sb sites and constructing Ti-centered octahedrons with surrounding Te atoms^8^. Such TiTe_2_ lamellae along with the Ti-centered octahedrons can preserve their ordering configurations even after high temperature RESET operations, which is believed to be responsible for the performance boost of the Ti_0.4_Sb_2_Te_3_ based PCM cell^7^,^8^.

The influence of Ti doping content (x) on the phase change properties of Ti_x_Sb_2_Te_3_ materials was also studied^9^,^10^. Although increasing Ti content can enhance 10-year data retention, reduce the volume change upon phase transition, improve interfacial adhesion ability, excessive Ti content (x ≈ 0.56, ∼10.1 at.%) causes Ti segregation which leads to poor endurance characteristic of the PCM cell^9^,^10^. This phenomenon may correlate to a low solid solubility of Ti atoms in the HEX-Sb_2_Te_3_ lattice, where only limited number of Ti-centered octahedrons could dispersedly distribute inside the quintuple-layered building blocks so as to preserve the ordering configuration^8^. To inhibit the Ti precipitation after repeated RESET-SET operations, it would be worthwhile to try to concomitantly raise the Te content of the non-stoichiometric Ti_x_Sb_2_Te_3_ materials. Thus, in this paper, we show the better electrical phase change properties based on a stoichiometric Ti_1_Sb_2_Te_5_ material. It is like a pseudobinary compound with 1 : 1 ratio of TiTe_2_ and Sb_2_Te_3_ components. Even containing higher Ti content (12.5 at.%), the Ti_1_Sb_2_Te_5_ based PCM cell has superior endurance characteristic over the Ti_0.56_Sb_2_Te_3_ based one. In addition, without significantly sacrificing the SET speed, the RESET energy of the Ti_1_Sb_2_Te_5_ based PCM cell is further lowered by 42∼47% compared to the Ti_0.4_Sb_2_Te_3_ based one. We argue that the richer concentration of TiTe_2_ lamellae in c-Ti_1_Sb_2_Te_5_ plays a major role in decreasing the RESET energy, meanwhile the lack of Ti-centered octahedrons resided in the quintuple-layered Sb_2_Te_3_ lattice may slow down the nucleation rate, however the increasing TiTe_2_ lamellae can act as structure-ordering template to enhance the crystal growth rate^11^. Accordingly, we believe that Ti_1_Sb_2_Te_5_ material is promising for realizing DRAM-like PCM application once advanced fabrication techniques being applied to further shrink the device dimension.

Figure 1 shows the temperature-dependent sheet resistance (*R*_s_) curves of as deposited TiTe_2_, Sb_2_Te_3_, Ti_0.4_Sb_2_Te_3_, and Ti_1_Sb_2_Te_5_ films upon *in situ* annealing with a heating rate of 10 °C/min. As the annealing temperature increases, a continuous decrease in *R*_s_ is observed for each film. Due to the partial crystallization during the sputtering process, compared to Ti_0.4_Sb_2_Te_3_ and Ti_1_Sb_2_Te_5_ films, both TiTe_2_ and Sb_2_Te_3_ films have smaller initial *R*_s_ and present a smooth decrement in *R*_s_. By comparison, one can observe the sudden drop in *R*_s_ occurs for Ti_0.4_Sb_2_Te_3_ and Ti_1_Sb_2_Te_5_ films when the temperature reaches *T*_c_ (both around 186 °C)^9^. The decrease in *R*_s_ with increasing temperature just before the onset of the crystallization indicates a semiconductor-like behavior. The temperature dependence for the *R*_s_ in a semiconductor can be expressed by *R*_s_ = *R*_s0_exp(−*E*_σ_/*kT*)^12^, where *R*_s0_ is a pre-exponential factor and *E*_σ_ is the activation energy for electrical conduction. The fitting results of *E*_σ_s of Ti_0.4_Sb_2_Te_3_ and Ti_1_Sb_2_Te_5_ are 0.11 eV and 0.13 eV, respectively. The activation energy of electrical transport is simply determined by half of the band gap *E*_σ_ = *E*_G_/2 + Δ*E*, where *E*_G_/2 is the distance from the Fermi level to the conduction band and Δ*E* is the depth of the trap states^13^. In the case of intrinsic conduction with equal amounts of electrons and holes, the Fermi level is situated at the middle of the band gap. Thus we can roughly estimate the optical band gaps (*E*_OP_s) of a-Ti_0.4_Sb_2_Te_3_ and a-Ti_1_Sb_2_Te_5_ to be ∼0.22 eV and ∼0.26 eV, respectively, both of which are quite smaller than that of a-Ge_2_Sb_2_Te_5_ (∼0.70 eV)^14^. Because the carrier density inside the semiconductor is proportional to exp(−*E*_G_/2*kT*), where *E*_G_ is the electrical band gap which is roughly identical to the *E*_OP_, a decrease in the band gap as the temperature approaching to the *T*_c_ will lead to the generation of a large number of carriers, which makes a major contribution to the quick drop in film resistivity^14^. On this view, one may roughly estimate that a-Ti_0.4_Sb_2_Te_3_ and a-Ti_1_Sb_2_Te_5_ could have quite faster crystallization (resistivity decrement) speed than that of a-Ge_2_Sb_2_Te_5_, and a-Ti_0.4_Sb_2_Te_3_ could be a little quicker than a-Ti_1_Sb_2_Te_5_.

Figure 2a compares the SET speed of Ge_2_Sb_2_Te_5_, Ti_0.4_Sb_2_Te_3_, and Ti_1_Sb_2_Te_5_ based PCM cells with the same size (BEC *D* = 190 nm), which has the same trend as aforementioned estimation. As the magnitude of applied voltage pulse reaches 1.3 V and 1.5 V, respectively, both the Ti_0.4_Sb_2_Te_3_ and Ti_1_Sb_2_Te_5_ cells show the SET speed of ∼6 ns. Apparently, under lower bias, the Ti_0.4_Sb_2_Te_3_ cell can complete the SET operation more quickly than the Ti_1_Sb_2_Te_5_ cell. In contrast, the SET operation of Ge_2_Sb_2_Te_5_ cell requires ∼75 ns at 1.6 V and ∼35 ns even at 2.1 V. In other words, one order of magnitude faster SET speed can still be achieved even by using the Ti_1_Sb_2_Te_5_ cell. Supplementary Information Figure S1 shows the cell resistance versus required time curves for SET operation of such three cells.

In terms of the RESET operation, even the Ti_0.4_Sb_2_Te_3_ (3.12 nJ) and Ti_1_Sb_2_Te_5_ (1.65 nJ) cells with *D* = 190 nm BEC have noticeable lower energies compared to the Ge_2_Sb_2_Te_5_ cells with smaller BEC (9.48 nJ for *D* = 130 nm BEC and 4.20 nJ for *D* = 80 nm BEC). More significant RESET energy reduction can be achieved on the Ti_0.4_Sb_2_Te_3_ (0.95 nJ) and Ti_1_Sb_2_Te_5_ (0.55 nJ) cells with *D* = 80 nm BEC. Namely, ∼78% and ∼87% of the energy have been saved via using the Ti_0.4_Sb_2_Te_3_ and Ti_1_Sb_2_Te_5_ cells (*D* = 80 nm BEC), respectively. Note that the Ti_1_Sb_2_Te_5_ cell achieves a substantial (42∼47%) RESET energy reduction on the basis of the Ti_0.4_Sb_2_Te_3_ cell. The shrinkage of BEC size also remarkably decreases the RESET current as shown in the Supplementary Information Figure S2. An ∼82% reduction of the RESET current is realized for both the Ti_0.4_Sb_2_Te_3_ and Ti_1_Sb_2_Te_5_ cells (∼0.5 mA) compared to that of Ge_2_Sb_2_Te_5_ cell (∼2.8 mA) with the same *D* = 80 nm BEC. Moreover, the RESET current of the Ti_1_Sb_2_Te_5_ cell (∼1.1 mA) is relatively smaller than that of the Ti_0.4_Sb_2_Te_3_ cell (∼1.3 mA) with the same *D* = 190 nm BEC. In addition to the improvements in RESET energy and current, the endurance characteristics of the Ti_1_Sb_2_Te_5_ cell (∼10^7^ cycles) is not inferior to that of the Ti_0.4_Sb_2_Te_3_ cell^7^, which is obviously far more better than that of the Ti_0.56_Sb_2_Te_3_ cell (<10^6^ cycles with severe fluctuation of the RESET state)^9^, as shown in the Supplementary Information Figure S3.

We used Raman spectroscopy (Fig. 3a) and *in situ* XRD (Fig. 3b) to characterize the c-Ti_1_Sb_2_Te_5_ phase. As a CdI_2_-like structure with space group P
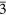
m1 space symmetry, c-TiTe_2_ has two Raman active modes produced entirely by the Te atoms for both the in-plane, *E*_*g*_ peak (∼122 cm^−1^), and out of plane, *A*_*1g*_ peak (∼143 cm^−1^), with the Ti atoms at rest^15^. Since there is no distinctive shoulder near ∼160 cm^−1^, the c-TiTe_2_ film can be considered as a stoichiometric compound without noticeable defects or impurities^16^. Because c-Sb_2_Te_3_ crystal belongs to the space group R
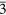
m, it has three Raman active modes, including two out of plane vibrations *A*_*1g*_(1) peak (∼69 cm^−1^) and *A*_*1g*_(2) peak (∼165 cm^−1^), and one in-plane vibration *E*_*g*_(1) peak (∼112 cm^−1^)^17^. Compared to the Raman curves of the c-TiTe_2_ and c-Sb_2_Te_3_ films, the c-Ti_1_Sb_2_Te_5_ film has five Raman active modes identically peaked corresponding to *E*_*g*_ and *A*_*1g*_ of TiTe_2_ and *A*_*1g*_(1), *A*_*1g*_(2), and *E*_*g*_(1) of Sb_2_Te_3_, respectively, as shown in Fig. 3a. The coexistence of c-TiTe_2_ and c-Sb_2_Te_3_ Raman active modes in c-Ti_1_Sb_2_Te_5_ no doubt shall originate from the two separated phases which is already observed in c-Ti_0.4_Sb_2_Te_3_^8^. In fact, our *in situ* XRD result of Ti_1_Sb_2_Te_5_ film clearly proves such phase separation phenomenon as shown in Fig. 3b, where both HEX-Sb_2_Te_3_ and HEX-TiTe_2_ diffraction peaks can be identified. Nevertheless we did not observe such distinct phase separation in Ti_0.4_Sb_2_Te_3_ through the same *in situ* XRD measurement^7^. The phase separation in Ti_0.4_Sb_2_Te_3_ occurs in nano-scale dimension^8^, where the TiTe_2_ lamellae segregate into no more than 10 nm-width belts and most of the TiTe_2_ lamellae (<2 nm in width) inlay with the Sb_2_Te_3_ quintuple-layered blocks. Due to the similar HEX lattice structures of TiTe_2_ and Sb_2_Te_3_, and also considering the lower doping content of Ti (∼7.4 at.%) in Ti_0.4_Sb_2_Te_3_, less concentration of the TiTe_2_ lamellae may result in undetected XRD signal. Note that the pure TiTe_2_ alloy has quite high *T*_m_ (>1200 °C)^18^ and the HEX-phase of its thin film can be maintained even at 700 °C (higher than the *T*_m_ ∼618 °C of Sb_2_Te_3_)^8^, thus there is no segregated Te or Ti phase being observed in c-Ti_1_Sb_2_Te_5_ as shown in Fig. 3b.

Since the Ti dopants in c-Ti_0.4_Sb_2_Te_3_ either segregate in the form of TiTe_2_ lamellae or construct Ti-centered octahedrons scattered in the quintuple-layered blocks of the Sb-Te lattice^8^, this non-stoichiometric composition can be chemically regarded as (TiTe_2_)_x_Ti_0.4−x_Sb_2_Te_3−2x_ (0 < x < 0.4), where (TiTe_2_)_x_ part corresponds to the TiTe_2_ lamellae and in Ti_0.4−x_Sb_2_Te_3−2x_ part the Ti_0.4−x_Te_0.8−2x_ accounts for the scattered Ti-centered octahedrons. The Ti_0.4-x_Sb_2_Te_3-2x_ part behaves more like the Sb-rich Sb-Te compound contributing to the faster recrystallization^8^. Extending this analysis to the stoichiometric Ti_1_Sb_2_Te_5_ = (TiTe_2_)_y_Ti_1-y_Sb_2_Te_5-2y_ (0 < y < 1), where (TiTe_2_)_y_ part also stands for the segregated TiTe_2_ lamellae, one can easily find that, in Ti_1−y_Sb_2_Te_5−2y_ part (=Ti_1−y_Te_2−2y_Sb_2_Te_3_), if Ti_1−y_Te_2−2y_ is assigned as the Ti-centered octahedrons accommodated in Sb-Te quintuple-layered blocks, it will be contradictory to keep the rest Sb-Te part in a ratio of 2 : 3 without Te deficiency. In other words, since there is no Ti or Te phase separation, it is more appropriate to describe the c-Ti_1_Sb_2_Te_5_ as (TiTe_2_)_1_(Sb_2_Te_3_)_1_ in which all the Ti atoms are supposed to be contained in the separated TiTe_2_ lamellae. Of course, this is a rough estimate, however, we may still get a reasonable inference that compared to the c-Ti_0.4_Sb_2_Te_3_, c-Ti_1_Sb_2_Te_5_ should have a higher concentration of the c-TiTe_2_ lamellae but a lower concentration of the “solid-solute” Ti-centered octahedrons.

Note that the quasi-two-dimensionalc-TiTe_2_ is semimetallic^19^ (see Fig. 1 also) with a quite low thermal conductivity (∼0.12 W/mK)^20^. The TiTe_2_ lamellae could act like the embedded nano-electrodes in c-Ti_1_Sb_2_Te_5_ to conduct the electrical current to the adjacent Sb_2_Te_3_ grains to generate Joule heat. The heat dissipation could be concomitantly refrained by those low thermal-conductive TiTe_2_ lamellae so as to effectively enhance the thermal efficiency of the RESET operation. Not surprisingly the c-Ti_1_Sb_2_Te_5_ (∼1/2 = 50.0% concentration of TiTe_2_ lamellae) based PCM cell can accomplish substantial reduction of the RESET energy compared to the c-Ti_0.4_Sb_2_Te_3_ (< ∼0.4/1.4 ≈ 28.6% concentration of TiTe_2_ lamellae) based PCM cell. On the contrary, the lack of survived Ti-centered octahedrons in Sb-Te rich amorphous matrix after RESET operation may slow down the nucleation rate for recrystallization process^8^,^9^, leading to a relatively slower SET speed for the Ti_1_Sb_2_Te_5_ based PCM cell as compared to that of the Ti_0.4_Sb_2_Te_3_ based one.

We also used the two-dimensional finite element method (FEM) to simulate and compare the RESET operations of the Ge_2_Sb_2_Te_5_, Ti_0.4_Sb_2_Te_3_, and Ti_1_Sb_2_Te_5_ based PCM cells, as shown in Fig. 4. The Joule heat is mainly generated in the phase change films. The thermal transfer obeys the standard heat conduction equation:^21^


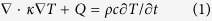


where *κ*, is the thermal conductivity, *c*, the specific heat, *ρ*, the density, *t*, the time, *T*, the temperature, and *Q*, the Joule heat per unit volume and per unit time, which is called the heat density. The key material parameters for FEM simulations include *κ* of c-Ge_2_Sb_2_Te_5_ (∼0.46 W/mK)^21^, c-Sb_2_Te_3_(∼0.78 W/mK)^22^, and c-TiTe_2_ (∼0.12 W/mK)^20^, *c* of c-Ge_2_Sb_2_Te_5_ (∼1.20 J/cm^3^K)^23^, c-Sb_2_Te_3_(∼1.02 J/cm^3^K)^24^, and c-TiTe_2_ (assumed to be ∼1.80 J/cm^3^K of c-TiSe_2_)^25^, and *ρ* of c-Ge_2_Sb_2_Te_5_ (∼6.2 g/cm^3^)^21^, c-Sb_2_Te_3_ (∼6.5 g/cm^3^)^26^, and c-TiTe_2_ (∼6.3 g/cm^3^)^27^. The phase change film layers of the models are divided into grid shape to represent the poly-crystalline morphology. Each grid denotes the small crystal grain. ∼29% and ∼50% of the grids in the c-Ti_0.4_Sb_2_Te_3_ and c-Ti_1_Sb_2_Te_5_ layers are randomly chosen to be the c-TiTe_2_ grains, respectively, as shown in Fig. 4b,c. Constant voltage pulse is applied to the axis-symmetric mushroom-type (T-shaped) cells. It can be observed the highest peak temperature is achieved in the Ti_1_Sb_2_Te_5_ based cell (Fig. 4c) while the Ge_2_Sb_2_Te_5_ based cell (Fig. 4a) has the lowest peak temperature. Apparently, more heat can be generated and confined in the phase change film layer as c-TiTe_2_ concentration increases, therefore lower energy is needed for the RESET operation.

In summary, the pseudobinary Ti_1_Sb_2_Te_5_ phase change alloy shows drastically decreased RESET energy, while increasing the SET speed, of the PCM cell compared to the Ge_2_Sb_2_Te_5_ based one. Without significantly reducing the SET speed as compared to the Ti_0.4_Sb_2_Te_3_ based PCM cell, nearly half of the RESET power can be saved on the Ti_1_Sb_2_Te_5_ based one. These improvements are achieved by introducing more nano-scale separated TiTe_2_ lamellae. We believe with more thermally stable TiTe_2_ lamellae the efficiency of electric conduction and heat inhibition could be greatly enhanced for the low-energy RESET operation. We expect the speed/power to be further increased/decreased significantly on thorough investigations of the (TiTe_2_)_n_(Sb_2_Te_3_)_m_ pseudobinary system and device dimension scaling techniques. In this regard, for example, a superlattice or multilayered structure constructed by alternate TiTe_2_/Sb_2_Te_3_ stacking film instead of the co-sputtering one will be a great help. It may also be possible to search topological superconducting properties on a finely-tuned TiTe_2_/Sb_2_Te_3_ superlattice sample.

## Methods

Ti_0.4_Sb_2_Te_3_ films were deposited by co-sputtering of pure Ti and Sb_2_Te_3_ targets. By adding an additional pure Te target, three-target co-sputtering technique was used to fabricate the Ti_1_Sb_2_Te_5_ films. Similarly, TiTe_2_ films were obtained by co-sputtering of pure Ti and Te targets. For Sb_2_Te_3_ and Ge_2_Sb_2_Te_5_ films, respective pure alloy target was used for sputtering. The compositions of all films were measured by X-ray fluorescence spectroscopy using a Rigaku RIX 2100 system. The temperature-dependent sheet resistance changing trends of TiTe_2_, Sb_2_Te_3_, Ti_0.4_Sb_2_Te_3_, and Ti_1_Sb_2_Te_5_ films with the same 150 nm thickness were studied by Linkam LMP 95 hot stage. For real-time observation of structure transition in Ti_1_Sb_2_Te_5_ film, vacuum *in situ* X-ray diffraction (XRD) measurement with a 20 °C/min heating rate was performed on 300-nm-thick film (deposited on Si substrate at room temperature) using PANalytical X’Pert PRO diffractometer with a Cu K*α* (λ = 0.15418 nm) radiation source. The diffraction data were collected in the 2*θ* range of 10°–60° with a scanning step of 0.02°. Raman spectroscopy (Thermo Fisher DXR) was performed on 300-nm-thick film samples at room temperature using an Ar^+^ laser (wavelength 532 nm) with ∼1 μm^2^ beam spot.

T-shaped PCM cells with diameter (*D*) = 190 (80) nm tungsten plug bottom electrode contact (BEC) were fabricated using 0.13 μm complementary metal-oxide semiconductor technology. In all the PCM cells, the thickness of the switching material films is around 170 nm. The 15-nm-thick TiN and 300-nm-thick Al films were used as top electrode for all cells. All the electrical measurements were performed by using the Keithley 2600C source meter (measuring cell resistance), the Tektronix AWG5002B pulse generator (generating voltage pulse with a minimum width of ∼6 ns), the homemade constant current driver (generating current pulse with a maximum magnitude of ∼10 mA), and the Tektronix 7054 digital phosphor oscilloscope (measuring transient voltage drop across the cell when current pulse is applied).

## Additional Information

**How to cite this article**: Ding, K. *et al*. Low-Energy Amorphization of Ti_1_Sb_2_Te_5_ Phase Change Alloy Induced by TiTe_2_ Nano-Lamellae. *Sci. Rep.* **6**, 30645; doi: 10.1038/srep30645 (2016).

## Supplementary Material

Supplementary Information

## Figures and Tables

**Figure 1:**
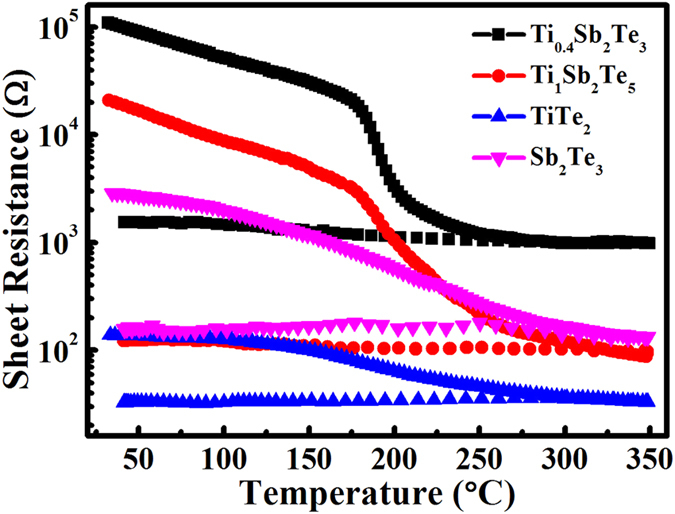
The sheet resistance (R_s_) as a function of *in situ* annealing temperature for the TiTe_2_, Sb_2_Te_3_, Ti_0.4_Sb_2_Te_3_, and Ti_1_Sb_2_Te_5_ films. The 150 nm thick films deposited on the SiO_2_/Si (1 0 0) substrate are measured with the *in situ* heating rate of 10 °C/min.

**Figure 2:**
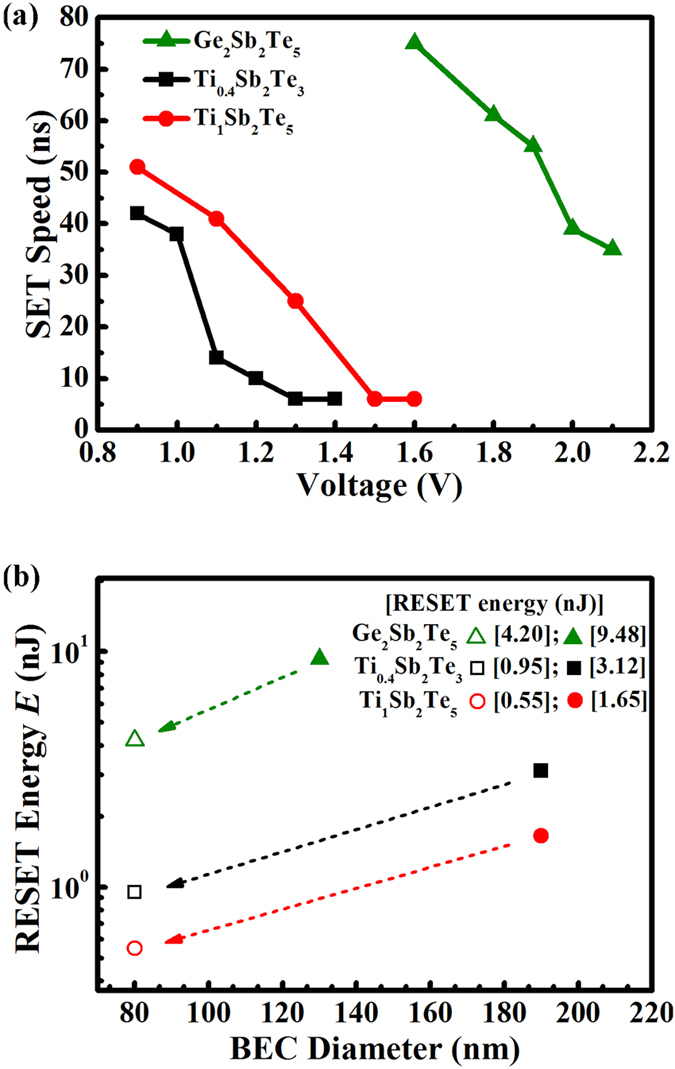
Comparisons of the SET operation speed and RESET energy. (**a**) SET operation speeds for the Ge_2_Sb_2_Te_5_, Ti_0.4_Sb_2_Te_3_, and Ti_1_Sb_2_Te_5_ based PCM cells with the same *D* = 190 nm W BEC. (**b**) RESET energy as a function of BEC diameter *D* for the Ge_2_Sb_2_Te_5_, Ti_0.4_Sb_2_Te_3_, and Ti_1_Sb_2_Te_5_ based PCM cells.

**Figure 3:**
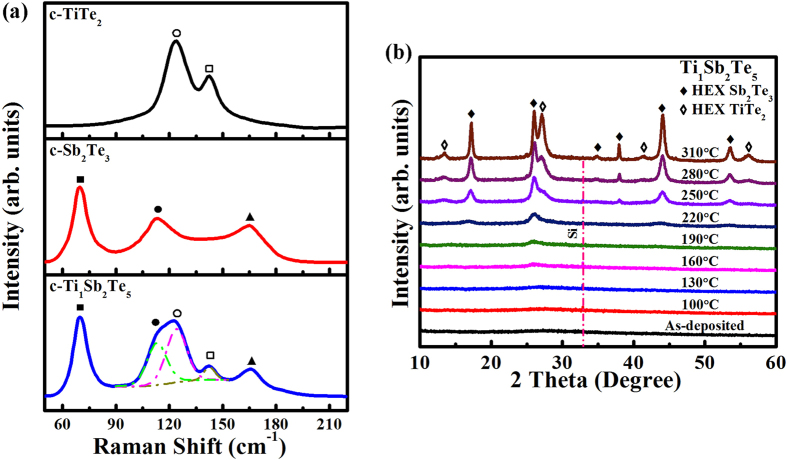
The Raman spectra of c-TiTe_2_, c-Sb_2_Te_3_, and c-Ti_1_Sb_2_Te_5_ and *in situ* XRD results of Ti_1_Sb_2_Te_5_. (**a**) Raman spectra of the c-TiTe_2_, c-Sb_2_Te_3_, and c-Ti_1_Sb_2_Te_5_ films. One can see two main *E*_*g*_ (○) and *A*_*1g*_ (□) phonon modes for c-TiTe_2_ film, and three main *A*_*1g*_(1) (■), *E*_*g*_(1) (●), and *A*_*1g*_(2) (▲) phonon modes for c-Sb_2_Te_3_ film. The c-Ti_1_Sb_2_Te_5_ film shows all the five phonon modes. (**b**) *In situ* XRD curves of Ti_1_Sb_2_Te_5_ film at different temperatures, where HEX lattice planes of Sb_2_Te_3_ (♦) and TiTe_2_ (◊) can be identified.

**Figure 4:**
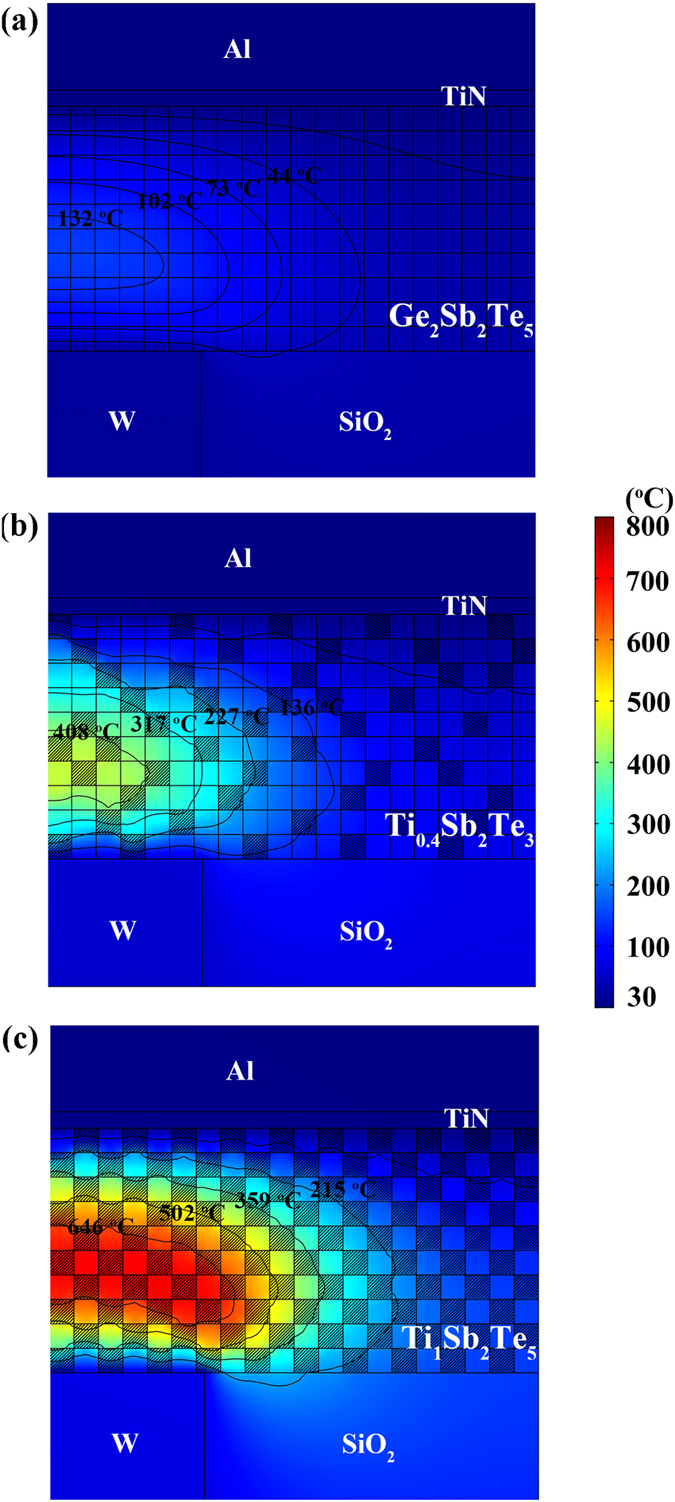
The two-dimensional finite element method simulations for the RESET operation. Simulated RESET temperature distributions in PCM cells with (**a**) c-Ge_2_Sb_2_Te_5_, (**b**) c-Ti_0.4_Sb_2_Te_3_, and (**c**) c-Ti_1_Sb_2_Te_5_ layers. In (**a**) all the square grids represent the small Ge_2_Sb_2_Te_5_ crystal grains, while in (**b**,**c**) the grids marked with slash lines stand for the TiTe_2_ crystal grains, and other non-marked ones are belonged to the Sb_2_Te_3_ crystal grains. The isothermal curves with corresponding temperatures in the PCM cells are also shown.
