# Supplementary material for: Low-Energy Amorphization of Ti1Sb2Te5 Phase Change Alloy Induced by TiTe2 Nano-Lamellae
Source: Sci Rep. 2016 Jul 29;6:30645. doi: 10.1038/srep30645 (PMC4965780; doi:10.1038/srep30645)
Supplement: Supplementary Information [file srep30645-s1.pdf]

Low-Energy Amorphization of $Ti_1Sb_2Te_5$ Phase Change Alloy Induced by $TiTe_2$ Nano-Lamellae

Keyuan Ding[1, 2], Feng Rao[1], Shilong Lv[1], Yan Cheng[1], Liangcai Wu[1], Zhitang Song[1]

[1]State Key Laboratory of Functional Materials for Informatics, Shanghai Institute of Micro-system and Information Technology, Chinese Academy of Sciences, Shanghai 200050, China.

[2]University of the Chinese Academy of Sciences, Beijing 100080, China.

Correspondence and requests for materials should be addressed to F. R. (email: fengrao@mail.sim.ac.cn).

Supplementary Information

Figure S1| Resistance versus required time (t_{SET}) for SET operation for (a) $Ge_2Sb_2Te_5$, (b) $Ti_{0.4}Sb_2Te_3$, and (c) $Ti_1Sb_2Te_5$ based phase change memory cells with the same diameter of 190 nm bottom electrode. The $Ge_2Sb_2Te_5$ cell requires t_{SET} = 75 ns at a 1.6 V voltage pulse to reach the low resistance (SET) state. A minimum time t_{SET} = 35 ns is obtained at a 2.1 V voltage pulse. While for the $Ti_{0.4}Sb_2Te_3$ cell, the SET state is reached for t_{SET} = 42 ns at a 0.9 V voltage pulse. A minimum time t_{SET} = 6 ns is obtained at a 1.3 V voltage pulse. In the case of the $Ti_1Sb_2Te_5$ cell, t_{SET} = 41 ns at a 1.1 V voltage pulse is required for achieving the SET state. A minimum time t_{SET} = 6 ns is obtained at a 1.5 V voltage pulse. It is obvious that both $Ti_{0.4}Sb_2Te_3$ and $Ti_1Sb_2Te_5$ cells have one order of magnitude faster SET operation speed than that of the $Ge_2Sb_2Te_5$ cell. Due to the limitation of the maximum output frequency (370 MHz) of our Tektronix AWG5002B pulse generator, the narrowest measurable square-wave voltage pulse is around 6 ns. Therefore, once the t_{SET} has reached 6 ns at certain voltage, further increasing the voltage pulse magnitude cannot reflect the fact that t_{SET} may be still decreased.[1] Thus, even when the voltage is increased to 1.4~1.5 V on the $Ti_{0.4}Sb_2Te_3$ cell and 1.6 V on the $Ti_1Sb_2Te_5$ cell, we cannot observe any decrease in t_{SET}.

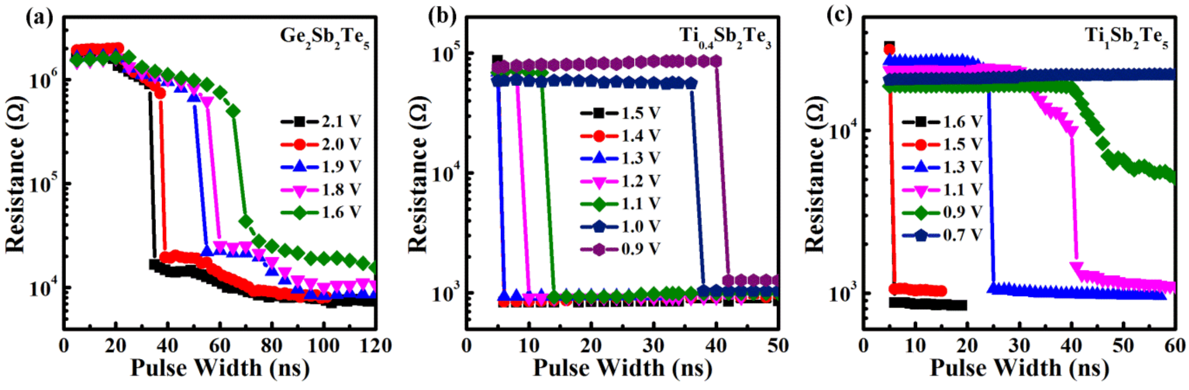

Figure S2| Resistance versus RESET current (I_{RESET}) curves with a fixed pulse width (*t*) of 1000 ns for $Ge_2Sb_2Te_5$, $Ti_{0.4}Sb_2Te_3$, and $Ti_1Sb_2Te_5$ based phase change memory cells with bottom electrode contact (BEC) of different diameters (*D*). Transient Reset voltage (U_{RESET}) across the cell is recorded once the RESET state is reached. The input RESET energy (*E*) can thus be calculated as $E = I_{RESET} \cdot U_{RESET} \cdot t$. The I_{RESET} and U_{RESET} values are indicated in the plot as marked by the arrows. The $Ge_2Sb_2Te_5$ cells require 9.48 nJ (D = 130 nm BEC) and 4.20 nJ (D = 80 nm BEC) RESET energy, respectively. While the $Ti_{0.4}Sb_2Te_3$ cells only need 3.12 nJ (D = 190 nm BEC) and 0.95 nJ (D = 80 nm BEC) for the RESET operation, respectively. Further RESET energy reduction can be achieved on the $Ti_1Sb_2Te_5$ cells (1.65 nJ for D = 190 nm BEC and 0.55 nJ for D = 80 nm BEC). The I_{RESET} of the $Ti_{0.4}Sb_2Te_3$ and $Ti_1Sb_2Te_5$ cells are also substantially reduced as compared to those of the $Ge_2Sb_2Te_5$ cells.

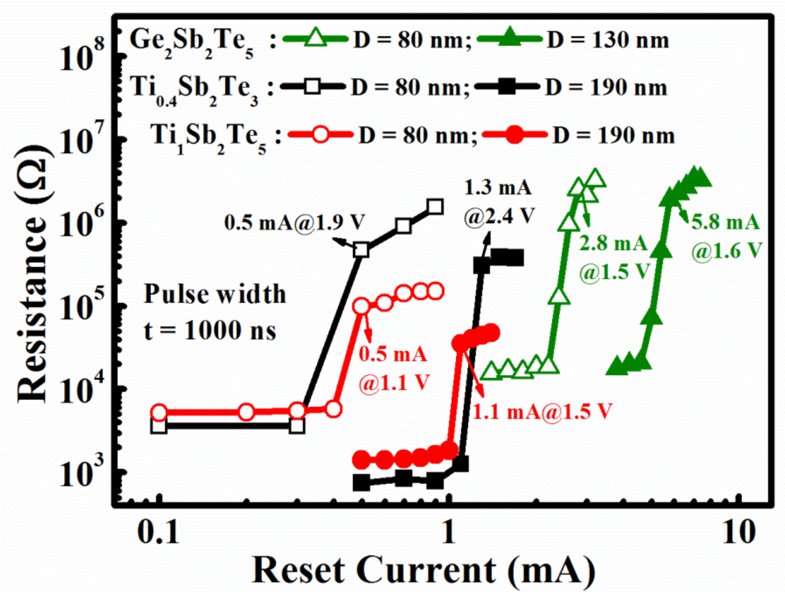

Figure S3| Endurance characteristics of the (a) $Ti_{0.56}Sb_2Te_3$ and (b) $Ti_1Sb_2Te_5$ phase change memory cells with the same diameter of 190 nm bottom electrode. The $Ti_{0.56}Sb_2Te_3$ cell apparently needs larger voltages to separate the SET and RESET states compared to those of the $Ti_1Sb_2Te_5$ cell. In contrast to the stable RESET state of the $Ti_1Sb_2Te_5$ cell, the RESET resistance begins to fluctuate after 10^5 SET-RESET cycles for the $Ti_{0.56}Sb_2Te_3$ cell. At ~10^6 SET-RESET cycles the $Ti_{0.56}Sb_2Te_3$ cell becomes invalid due to the SET-stuck failure which may be caused by excessive Ti phase separation.[2] In comparison, the $Ti_1Sb_2Te_5$ cell can repeatedly perform SET-RESET operations up to 10^7 cycles with quite stable high/low resistance states, which is as good as that of the $Ti_{0.4}Sb_2Te_3$ cell.[2]

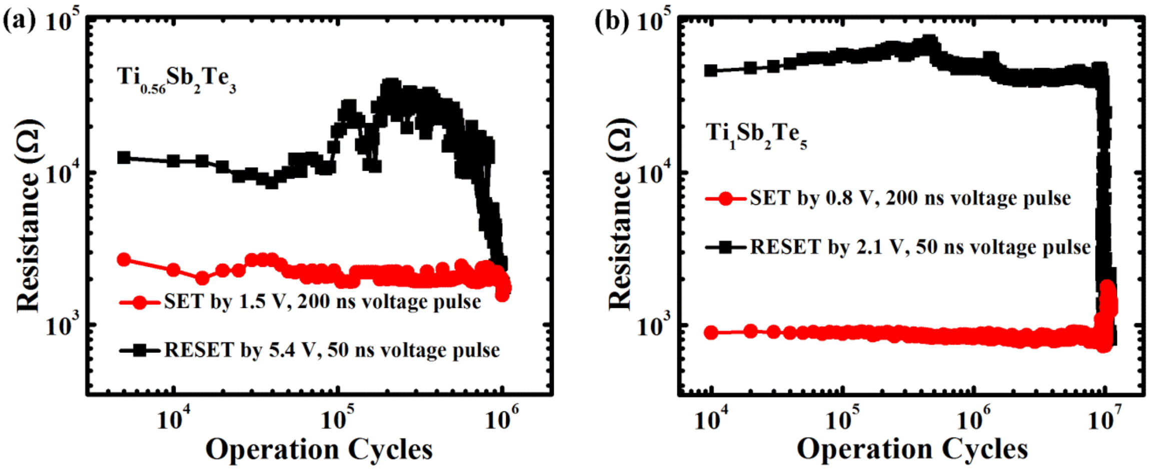

Supplementary Information References

(1) Wang, W. J. *et al*. Enabling Universal Memory by Overcoming the Contradictory Speed and Stability Nature of Phase-Change Materials. *Sci. Rep.* **2**, 360 (2012).

(2) Xia, M. J. *et al*. Ti−Sb−Te Alloy: A Candidate for Fast and Long-Life Phase-Change Memory. *ACS Appl. Mater. Inter*. **7**, 7627−7634 (2015).
